# In vitro anticancer activity of *Scrophularia amplexicaulis* extracts on MCF-7 and WEHI-164 cell line

**DOI:** 10.17179/excli2020-2737

**Published:** 2020-09-22

**Authors:** Samira Valiyari, Elham Beiranvand, Azin Samimi, Saeid Yaripour, Behzad Baradaran, Abbas Delazar, Mehdi Forouzesh

**Affiliations:** 1 Legal Medicine Research Center, Legal Medicine Organization, Tehran, Iran; 2 Department of Biotechnology, Pasteur Institute of Iran, Tehran, Iran; 3 Legal Medicine Research Center, Legal Medicine Organization, Ahvaz, Iran; 4 Department of Drug and Food Control, Faculty of Pharmacy, Tehran University of Medical Sciences, Tehran, Iran; 5 Immunology Research Center, Tabriz University of Medical Sciences, Tabriz, Iran; 6 Department of Pharmacognosy, Faculty of Pharmacy, Tabriz University of Medical Sciences, Tabriz, Iran

**Keywords:** Scrophularia amplexicaulis, anticancer, cytotoxicity, apoptosis

## Abstract

*Scrophularia amplexicaulis* is an Iranian endemic plant belonging to the Scrophulariaceae family, which is used in traditional medicine to treat many diseases. The aim of this study was to evaluate the *in vitro* anticancer activity of *S. amplexicaulis* extracts against human breast carcinoma (MCF-7) and mouse fibrosarcoma (WEHI-164) cell lines. The ground aerial parts of *S. amplexicaulis* were soxhlet-extracted with n-hexane, dichloromethane and methanol. MTT assay exhibited that dichloromethane and methanol extracts remarkbly inhibited the growth of MCF-7 and WEHI-164 cancer cells in a dose-and time-dependent manner with little cytotoxicity on normal cell line HUVEC. Cell death ELISA, TUNEL assay, and the cleavage of poly ADP-ribose polymerase (PARP) uncovered that the cytotoxic effects of dichloromethane and methanol extracts were attributed to apoptosis in cancerous cells. Furthermore, quantitative real-time PCR revealed significant increases in the mRNA expression levels of p-53, caspase-3, caspase-9, Bax, and also a decrease in Bcl-2 expression. These results suggested that the extracts mainly induced apoptosis via a mitochondria-mediated intrinsic pathway. Notably, dichloromethane extract had higher cytotoxic and apoptotic activities than that of methanol extract, against both cancer cell lines, particularly MCF-7 cells. Our results indicate that *S. amplexicaulis* may serve as a promising source of potent agents for the treatment of human cancers.

## Introduction

Nowadays cancer is a crucial public health problem and one of the leading causes of death throughout the world. Approximately 70 % of cancer related deaths occur in developing countries. The International Agency for Research on Cancer (IARC) estimated that the annual number of new cancer cases would rise to an estimated 21.7 million in 2030 along with 13.0 million deaths (Wilkes, 2018[39]; Dalton et al., 2019[6]). Among the various strategies for cancer treatment, chemotherapy is often known as the most widely used method to prevent and treat cancer. However, the current cancer chemotherapeutic drugs have severe side effects and acquired drug resistances (Alfarouk et al., 2015[3]; Srivastava et al., 2016[35]). Therefore, there is an imperative interest to find new effective and less-toxic therapeutic agents against cancer.

Plants have great potential to provide chemoprotective effects against cancer and over 60 % of anti-cancer drugs are derived from natural sources including plants, microorganisms and marine organisms (Sultana et al., 2014[37]; Fridlender et al., 2015[11]; Dey et al., 2019[9]). Plant-derived anticancer drugs such as taxol from *Taxus brevifolia* L., camptothecin from *Camptotheca acuminata*, Decne., vinca alkaloids from *Catharanthus roseus *(L.) G. Don and podophyllotoxin from *Podophyllum peltatum* L. are well-known and extensively used in clinical trial (Iqbal et al., 2017[15]; Kuruppu et al., 2019[18]). 

Different species of medicinal plants are shown to exert remarkable anticancer activity through apoptosis without causing undesired side effects (Safarzadeh et al., 2014[34]; Murthy and Alice, 2018[23]). Apoptosis, programmed cell death, is a vital component of various processes comprising normal cell turnover, embryonic development, immune system regulation, tissue homeostasis and chemical induced cell death. Dysfunction of the apoptotic cell death is powerfully associated with the cancer initiation, progression, metastasis and treatment resistance. Hence, it is a validated strategy to evaluate traditional medicinal plants for their apoptosis inducing potential in cancer cells (Pistritto et al., 2016[32]; Pfeffer and Singh, 2018[30]; Jan and Chaudhry, 2019[16]). 

*Scrophularia* is regarded as one of the large genera of the family Scrophulariaceae. This family comprises about 3000 species and 220 genera which are widely distributed in mountainous regions of the world, especially in Asia and North America (Giessrigl et al., 2012[13]; Pasdaran and Hamedi, 2017[28]). Since ancient times, diffrent *Scrophularia* species have been used in traditional medicines to treat fever, wounds, erythema, eczema, psoriasis, inflammatory diseases, mouth dryness, goiter, constipation, fistula and infectious diseases (Stevenson et al., 2002[36]; Park et al., 2009[26]). Apart from these activities, anticancer properties of different extracts from several *Scrophularia *species against various cancer models have been reported in the literature (Orangi et al., 2016[24]; Pasdaran and Hamedi, 2017[28]). Phenylpropanoid glycosides and iridoids are the main chemical compounds of *Scrophularia* genus which can lead to aforementioned therapeutic effects (de Santos Galíndez et al., 2002[7]; Bermejo et al., 2002[5]; Dıaz et al., 2004[10]).

*Scrophularia amplexicaulis *Benth. is one of the Iranian endemic species of Scrophulariaceae family. Phytochemical analysis of the aerial parts of this species revealed the presence of two phenylalkanoid glycosides, salidroside and verbascoside, and two iridoid glycosides, scropolioside D and scrophuloside B4 (Pasdaran et al., 2016[27]). The lack of any information about the anticancer activity of *S. amplexicaulis* prompted us to investigate the cytotoxic effects of *S. amplexicaulis *extracts against MCF-7 and WEHI-164 cancer cells and determine their underlying molecular mechanisms. The mouse firosarcoma cell line WEHI-164 was selected to confirm the aforementioned effect with the aim of performing further *in vivo *studies.

## Materials and Methods

### Reagents and cell lines

n-hexane, dichloromethane (DCM), methanol (MeOH), and dimethyl sulphoxide (DMSO) were of analytical grade and were purchased from Merck (Darmstadt, Germany). RPMI-1640 medium, fetal bovine serum (FBS), penicillin-streptomycin (100 units/mL penicillin and 100 μg/mL streptomycin), (3-[4, 5-dimethylthiazol-2-yl]-2,5-diphenyltetrazolium bromide (MTT), trypan-blue, paraformaldehyde, and ethylenediaminetetraacetate (EDTA) were obtained from Sigma Aldrich (MO, USA). Trypsin-EDTA (0.25 %) was obtained from Gibco (Paisley, UK). Cell death detection ELISA^plus^ kit, terminal deoxynucleotide transferase dUTP nick end labeling (TUNEL) assay kit, high pure RNA isolation kit, and protease inhibitor cocktail were purchased from Roche (Mannheim, Germany). RevertAid^TM ^first strand synthesis kit was obtained from Fermentas (MA, USA). SYBR Green PCR Master Mix was purchased from Applied Biosystems (CA, USA). Designed primers were purchased from Bioneer (Daejeon, Korea). Enhanced chemiluminescence (ECL) advance Western blotting detection kit was purchased from GE Healthcare (Little Chalfont, UK). Antibodies against poly ADP-ribose polymerase (PARP), β-actin, and rabbit IgG conjugated with horseradish peroxidase (HRP) were obtained from Abcam (MA, USA). Human breast cancer cell line MCF-7, mouse fibrosarcoma cell line WEHI-164, and normal human umbilical vein endothelial cells (HUVEC) were purchased from National Cell Bank of Iran (Pasteur Institute, Tehran, Iran).

### Preparation of extracts

The aerial parts of *S. amplexicaulis *were harvested from Sahand mountain, located in East Azerbaijan province, Iran. The plant was authenticated and a voucher specimen (2817) has been deposited at the Herbarium of Pharmacognosy Department, Faculty of Pharmacy, Tabriz University of Medical Sciences, Iran. The air-dried powdered aerial parts of *S. amplexicaulis *(1800 g) were subjected to Soxhlet apparatus and extracted sequentially with organic solvents n-hexane, dichloromethane (DCM), and methanol (MeOH). The extract solutions were then concentrated under reduced pressure using a rotary evaporator (Heildolph, Germany) at 45 °C. Afterward, 20 mg of each extract was separately dissolved in 100 µL of DMSO, diluted with RPMI-1640 medium and sterilized with 0.22 μm syringe filters (Nunc, Denmark). Finally, *S. amplexicaulis *extracts were stored at 4 °C for further biological experiments.

### Cell culture

The cells were grown in RPMI-1640 medium supplemented with 10 % FBS, 100 U/mL penicillin, and 100 µg/mL streptomycin under a humidified 5 % CO_2_ atmosphere at 37 °C. After 80 % confluence, the cells were detached by 0.25 % Trypsin-EDTA solution for the passage. 

### MTT assay

The cytotoxic activity of n-hexane, DCM and MeoH extracts of *S. amplexicaulis *on MCF-7, WEHI-164, and HUVEC cells was examined by MTT assay. Briefly, the cells in logarithmic growth phase were seeded into 96-well plates (Nunc, Denmark) at a density of 1× 10^4^ cells/well for 24 h at 37 °C with 5 % CO_2_ and treated with different concentrations of extracts (10, 20, 50, 100, 150, 200, 300, 400 µg/mL) for 12, 24 and 48 h. Untreated cells and cells exposed to paclitaxel (0.5 µg/mL) served as negative and positive control, respectively. Then, 20 μL of MTT solution (5.0 mg/mL) was added to the cells and incubated for 4 h at 37 °C. Subsequently, the culture medium was removed from each well and replaced with 200 µL DMSO and 50 µL Sorenson buffer. After 30 min of incubation at 37 °C, the absorbance was measured at 570 nm and a reference wavelength of 630 nm using a microplate ELISA reader (BioTeck, Germany). 

### Dye exclusion assay

The number of viable cells in a cell suspension was determined by trypan blue exclusion assay. Briefly, 1× 10^4 ^cells/well were dispensed into 96 well-plates and incubated for 24 h at 37 °C. The cells were then treated with same different concentrations of *S. amplexicaulis* extracts for 24 h. Untreated cells and cells exposed to paclitaxel (0.5 µg/mL) served as negative and positive control, respectively. At the end of incubation, the cells were trypsinized, washed twice with phosphate-buffered saline (PBS) and the resultant pellets were resuspended in 0.5 mL of PBS. Thereafter, equal volumes of 0.4 % trypan blue dye and cell suspensions were mixed and the number of viable and dead cells was counted using a hemocytometer (Weber, U.K.).

### Cell death detection

Apoptosis and necrosis was assessed by Cell Death Detection ELISA^plus^ kit according to the manufacturer's protocol. The cells were cultured at a density of 1×10^4 ^cells/well into 96-well plates for 24 h at 37 °C and then treated with DCM and MeOH extracts of *S. amplexicaulis *at their 48-h IC_50_ (50 % inhibitory concentration) concentrations for 24 h. Untreated cells were used as a negative control. Thereafter, the cells were trypsinized, centrifuged (1500 rpm for 5 min), the supernatants and lysates were collected and transferred into the microtiter plates coated with anti-histone antibody. After 90 min of incubation, the plates were rinsed three times with washing buffer, anti-DNA antibodies conjugated with horseradish peroxidase (HRP) were added and incubated for 2 h at room temperature (RT). Then, the plates were washed again and incubated with peroxidase substrate for 20 min at RT. The final absorbance at 405 nm was determined by a microplate ELISA reader (BioTeck, Germany).

### TUNEL assay

DNA fragmentation, a prominent hallmark of apoptosis, was detected using TUNEL assay kit following the manufacturer's instructions. Briefly, cells (2×10^5 ^cells/well) were seeded in 6-well chamber slides (Nunc, France) and incubated for 24 h at 37 °C. Following treatment with 48-h IC_50_ concentrations of DCM and MeOH extracts for 24 h, the treated and untreated cells (negative control) were fixed with 4 % (w/v) paraformaldehyde solution in PBS (pH 7.4) for 1 h at RT. The slides were then washed twice with PBS and incubated with blocking solution (3 % H_2_O_2_ in methanol) for 10 min at the same temperature. Subsequently, the cells were washed again and permeabilized with 0.1 % Triton X-100 in 0.1 % sodium citrate for 2 min on ice. After rinsing in PBS, 50 µl of terminal deoxynucleotide transferase (TdT) reaction mixtures was added to the cells and incubated for 1 h at 37 °C. The cells were then washed three times with PBS and incubated with 50 µl Streptavidin HRP solution for 30 min at 37 °C. Finally, the cells were stained with 100 μl of DAB substrate for 10 m in a dark condition and evaluated under a light microscope (Nikon, Japan).

### Western blotting

The cells were grown at a density of 1×10^6 ^cells/well in 6 well-plates and treated with 48-h IC_50_ concentrations of DCM and MeOH extracts for 24 h. Afterward, the treated and untreated cells (negative control) were harvested and lysed in lysis buffer (1 % Triton X-100, 1 M HEPES, 5 M NaCl and 0.5 M EDTA) supplemented with a protease inhibitor cocktail. Protein concentrations were determined using the Bradford method based on the bovine serum albumin standard. Equal amounts of protein were fractioned by 12 % sodium dodecyl sulfate-polyacrylamide gel electrophoresis (SDS-PAGE) and transferred onto polyvinylidene difluoride (PVDF) membranes (Roche, Germany). The membranes were then blocked with 4 % skim milk in Tris-buffered saline Tween-20 (TBST) for 2 h, incubated with anti-PARP (1:500 primary antibody overnight at 4 °C, washed three times with TBST, and incubated with HRP conjugated anti-rabbit IgG secondary antibody (1:1000) for 2 h at RT. After three washes with TBST, the protein bands were developed on X-ray film using ECL advance Western blotting detection kit. 

### Real-time PCR 

Following treatment, total RNA of treated and untreated cells (negative control) were extracted using a high pure RNA isolation kit. RNA samples were reverse transcribed into cDNA by RevertAid^TM ^first strand synthesis kit for quantitative real-time polymerase chain reaction (qRT-PCR) according to the manufacturer's protocol. Primers for the target genes (p-53, caspase-3, caspase-9, Bax, and Bcl-2) and the reference housekeeping gene β-actin were designed using the Primer-Blast tool from the NCBI website online (http://www.ncbi.nlm.nih.gov/tools/primer- blast/index) and their sequences summarized in Table 1(Tab. 1). qRT-PCR was performed using an ABI7900HT Sequence Detection System (Applied Biosystems, USA) with SYBR Green PCR Master Mix and designed primers. Sequentially, qRT-PCR conditions were 95 °C for 5 min, 40 cycles of at 95 °C for 15 s, 57 °C for 30 s and 60 °C for 1 min. The expression levels of target genes were normalized to β-actin and the relative fold changes were calculated using the ΔΔCt method (Livak and Schmittgen, 2001[19]).

### Statistical analysis

The data are expressed as the mean ± SD of three individual experiments performed in triplicate. The data were analyzed using IBM SPSS Statistics 20 software. Statistical analysis was performed by two-way ANOVA followed by Duncan's new multiple range test and *P* < 0.05 was considered statistically significant. The IC_50 _values were determined from the dose-response curves using GraphPad Prisms 6.01 software.

## Results

### Cytotoxic effects of S. amplexicaulis extracts on cancer cells

**Figure 1:**
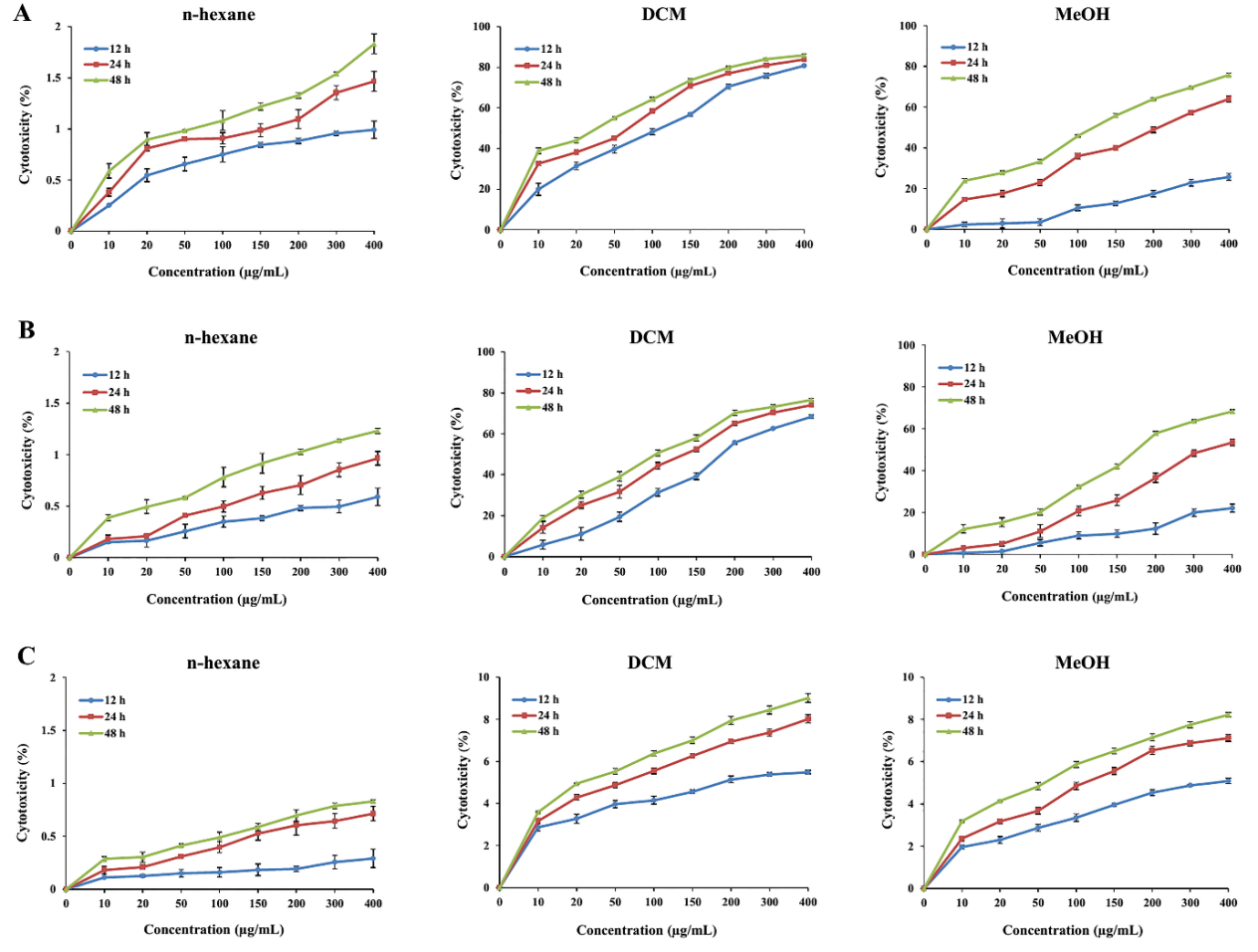
Cytotoxic effects of *S. amplexicaulis* extracts on (A) MCF-7, (B) WEHI-164, and (C) HUVEC. The cells were treated with different concentrations of extracts for 12, 24 and 48 h. Cytotoxicity was measured by MTT assay. Data are presented as the mean ± SEM of three independent experiments.

**Figure 2:**
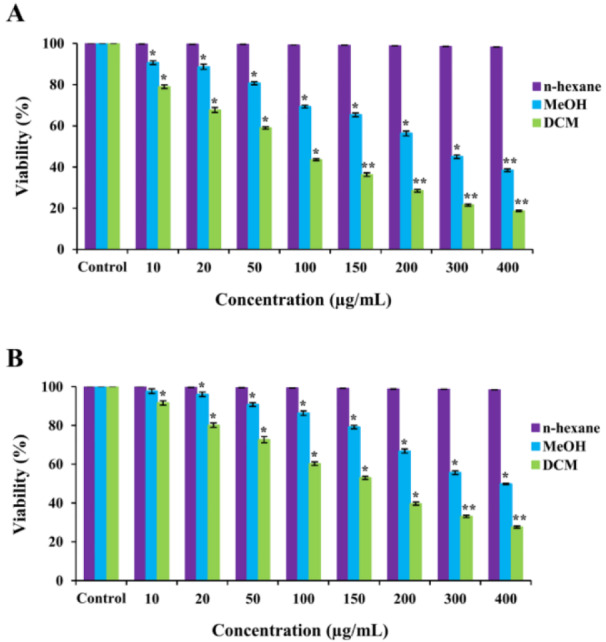
Cell viability of (A) MCF-7 and (B) WEHI-164. The cells were treated with different concentrations of extracts for 24 h. Viability of treated cells was determined by trypan blue exclusion assay. Data are presented as the mean ± SEM of three independent experiments. *P < 0.05, **P < 0.01 compared with the untreated control.

To assess the effects of *S. amplexicaulis* extracts on cell growth, MTT and trypan blue assays were performed. As shown in Figure 1A-B(Fig. 1), DCM and MeOH extracts exhibited significant dose- and time-dependent cytotoxic activity against MCF-7 and WEHI-164 cancer cell lines compared to the untreated control cells (*P* < 0.001). However, no growth inhibition was observed following treatment with n-hexane extract. Furthermore, DCM extract inhibited the growth of cancer cells more powerfully than did the MeOH extract (*P* < 0.01). Considering all IC_50_ values reported in Table 2(Tab. 2), MCF-7 cells were more sensitive to growth inhibitory effects of DCM and MeOH extracts compared with WEHI-164 cells at all tested time points. Interestingly, DCM and MeOH extracts revealed little cytotoxicity toward normal cell line HUVEC (IC_50_ > 400 µg/mL) (Figure 1C(Fig. 1)). Microscopic cell count using a hemocytometer showed a remarkable decrease in the number of viable MCF-7 and WEHI-164 cells following exposure to DCM and MeOH extracts (Figure 2A-B(Fig. 2)). In addition, paclitaxel decreased the viability of MCF-7 and WEHI-164 cells to 59.2 % ± 0.4 and 83.5 % ± 0.7 in comparison to the control cells, respectively (data not shown).

### Effects of S. amplexicaulis extracts on the induction of apoptosis

**Figure 3:**
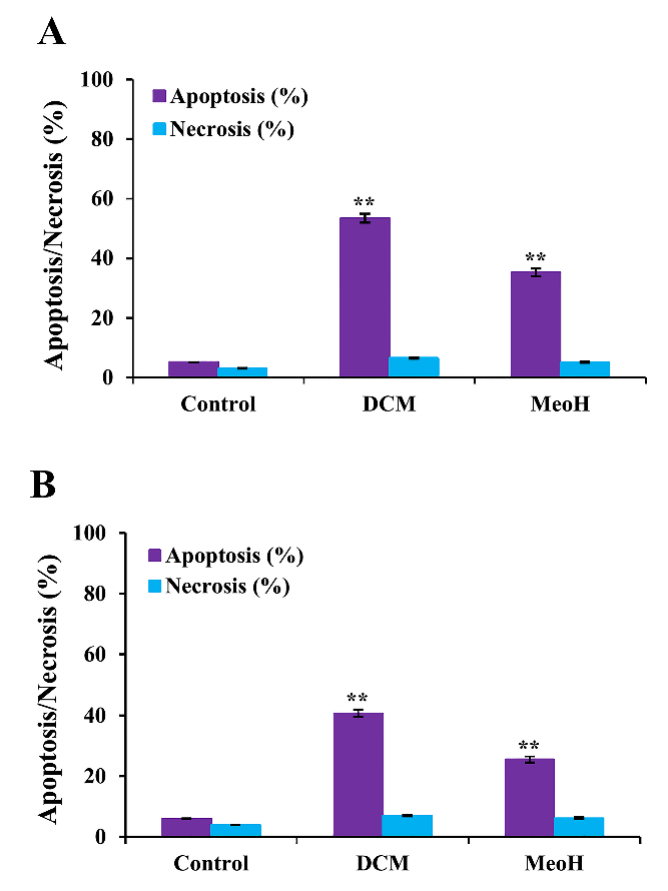
The percentage of apoptosis and necrosis in (A) MCF-7 and (B) WEHI-164 were evaluated by cell death detection ELISA kit. The cells were treated with 48-h IC_50_ concentrations of DCM and MeOH extracts of *S. amplexicaulis *for 24 h. Data are presented as the mean ± SEM of three independent experiments. **P < 0.01 compared with the untreated control.

**Figure 4:**
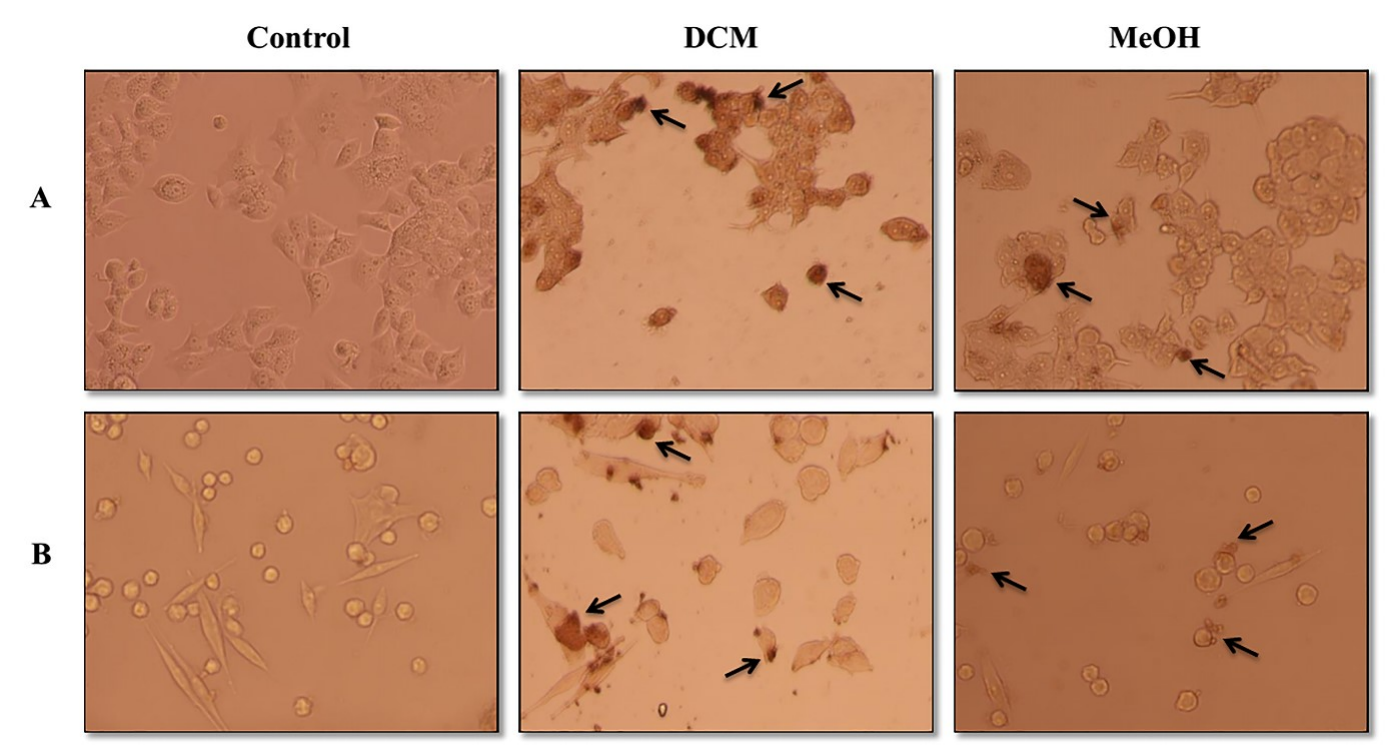
Apoptotic morphological alterations in (A) MCF-7 and (B) WEHI-164 were detected by TUNEL assay and light microscopy (×200). The cells were treated with 48-h IC_50_ concentrations of DCM and MeOH extracts of *S. amplexicaulis *for 24 h. Arrows indicate the apoptotic cells.

**Figure 5:**
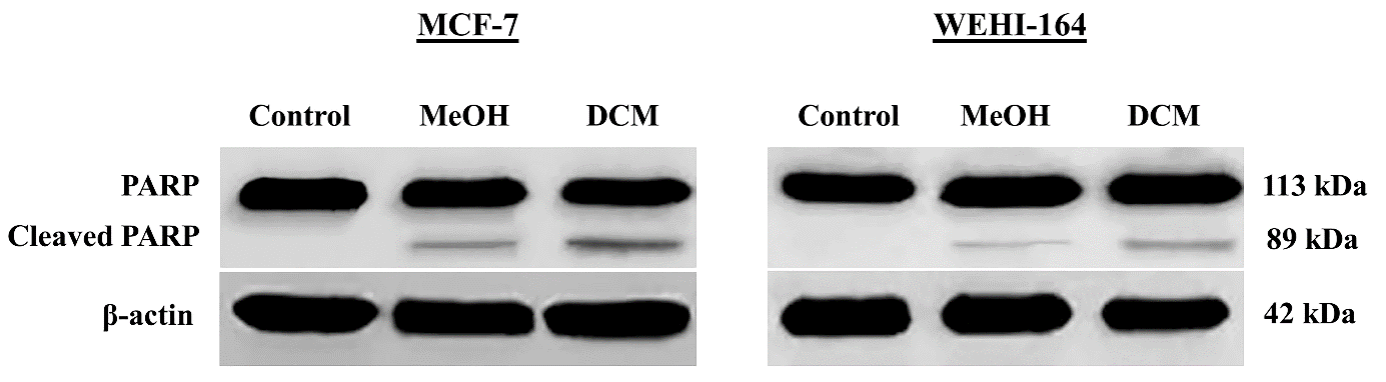
Effects of DCM and MeOH extracts of *S. amplexicaulis* on PARP cleavage in MCF-7 and WEHI-164. The cells were treated with 48-h IC_50_ concentrations of extracts for 24 h, and then the cleavage of PARP was determined using Western blotting. β-actin was used as a loading control.

To investigate whether DCM and MeOH extracts of *S. amplexicaulis *exhibit cytotoxicity due to apoptosis or necrosis, we used cell death detection ELISA kit. After 24 h of treatment with 48-h IC_50 _concentrations of DCM extract, the apoptosis rate significantly increased from 3.8 % and 2.9 % in the control cells to 53.5 % and 40.7 % in MCF-7 and WEHI-164 cancer cells, respectively (*P* < 0.01) (Figure 3A-B(Fig. 3)). When the cells were treated with 48-h IC_50_ concentrations of MeOH extract for 24 h, the percentages of apoptosis were 35.2 % and 25.5 % in MCF-7 and WEHI-164 cells, respectively (Figure 3A-B(Fig. 3)). However, DCM and MeOH extracts did not show any apoptotic or necrotic death in HUVEC normal cells (data not shown). Notably, our results implied that DCM extract induced a higher rate of apoptosis than that of MeOH extract in both cancer cell lines, particularly MCF-7 cells. Furthermore, TUNEL assay was performed to identify the presence of DNA fragmentation and confirm the induction of apoptosis in cells treated with DCM and MeOH extracts of *S. amplexicaulis*. As illustrated in Figure 4A-B(Fig. 4), the cells displayed dark brown stained nuclei following exposure to DCM and MeOH extracts, whereas no visible coloration was detected in the untreated control cells. We further examined the expression level of PARP cleavage in MCF-7 and WEHI-164 cells by Western blotting. Upon treatment with DCM and MeOH extract for 24 h, the expression of cleaved PARP was detected in both cancer cell lines (Figure 5(Fig. 5)). Overall, the results indicated that the cytotoxicity of DCM and MeOH extracts of *S. amplexicaulis* against MCF-7 and WEHI-164 cells is mainly mediated by apoptosis induction.

### Effect of S. amplexicaulis extracts on the mRNA expression of apoptotic-associated genes

**Figure 6:**
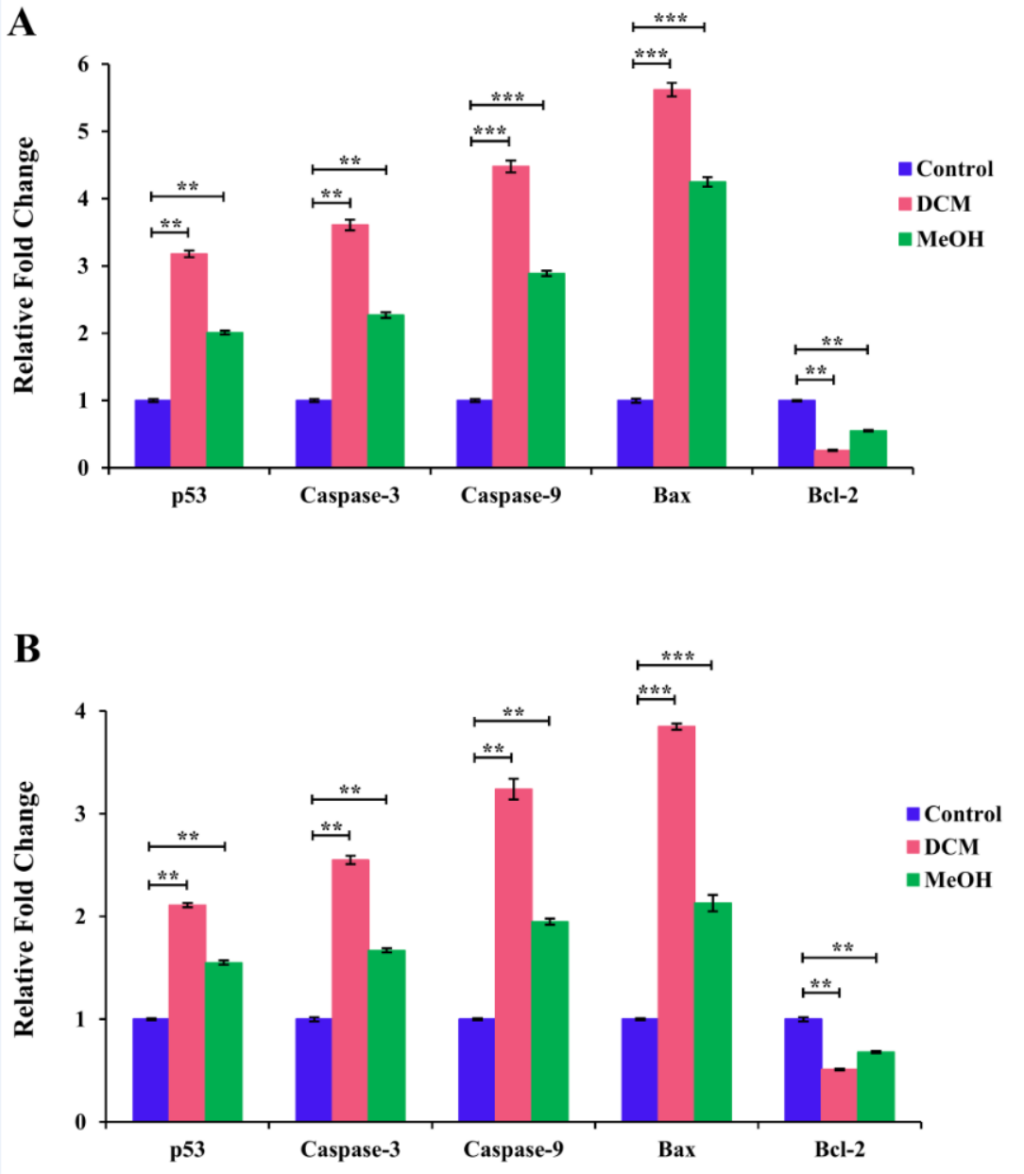
Relative mRNA expression levels of apoptotic-associated genes in (A) MCF-7 and (B) WEHI-164 were assessed by qRT-PCR. The cells were treated with 48-h IC_50_ concentrations of DCM and MeOH extracts of *S. amplexicaulis *for 24 h. Data are presented as the mean ± SEM of three independent experiments. **P < 0.01, ***P < 0.001 compared with the untreated control.

In order to evaluate the mRNA expression levels of apoptotic-associated genes including p-53, caspase-3, caspase-9, Bax, and Bcl-2 in MCF-7 and WEHI-164 cancer cells, qRT-PCR was carried out. As shown in Figure 6A-B(Fig. 6), treatment with 48-h IC_50_ concentrations of DCM extract for 24 h led to significant enhancement in the mRNA expression levels of p-53, caspase-3, caspase-9, and Bax 3.2, 3.6, 4.5, and 5.6 fold in MCF-7 cells and 2.1, 2.5, 3.2, and 3.9 fold in WEHI-164 cells, respectively, when compared with the control cells (*P* < 0.01 and *P* < 0.001). Besides that, MeoH extract treatment could significantly increase the mRNA levels of p-53, caspase-3, caspase-9, and Bax 2.0, 2.3, 2.9, and 4.3-fold in MCF-7 cells and 1.6, 1.7, 2.0, and 2.2-fold in WEHI-164 cells. In contrast, the expression level of Bcl-2 mRNA significantly decreased by about 6.8 and 2.1-fold in MCF-7 cells and 1.8 and 1.4-fold in WEHI-164 cells treated with DCM and MeOH extracts, respectively (*P* < 0.01 and *P* < 0.001). These results are in line with our previous findings demonstrating a more alteration in the mRNA expression levels of the apoptotic-associated genes for MCF-7 cells treated with DCM extract compared to all other groups (*P* < 0.01). The results obtained herein suggest that DCM and MeOH extracts of *S. amplexicaulis* can induce apoptosis in MCF-7 and WEHI-164 cells through the mitochondrial pathway.

## Discussion

Over the last few years, medicinal plant-based therapeutics have gained great attention in the treatment of cancer as they are more cost effective, natural, readily available and exhibit fewer undesirable side effects. In addition, the potential of plant-derived compounds to induce cellular apoptosis constitute a key event in anticancer activities of medicinal plants (Desai et al., 2008[8]; Maqsood et al., 2018[20]; Ahmed et al., 2019[2]). Herein, we performed a pilot study to firstly examine the cytotoxic and apoptotic effects of various extracts of *S. amplexicaulis* on cancer cell lines MCF-7, WEHI-164 and normal cell line HUVEC. The results demonstrated that DCM and MeOH extracts dose- and time-dependently inhibited the growth of MCF-7 and WEHI-164 cancer cells with little harmful effects on normal HUVEC cells, which highlight the selective cytotoxicity of these extracts. Furthermore, n-hexane extract did not show any inhibitory effects against cancer and normal cells. It is also worth mentioning that DCM extract exerted a cytotoxic activity more superior to that of MeOH extract at all tested time points. Thus, it could be concluded that high level of bioactive compounds of* S. amplexicaulis* may be in DCM extract. The other *Scrophularia *species such as *Scrophularia atropatana*, *Scrophularia oxysepala*, and *Scrophularia frigida* have also been reported to suppress the growth of breast cancer cells generally in a time- and dose- dependent way (Valiyari et al., 2013[38]; Goldar et al., 2016[14]; Safarzadeh et al., 2017[33]).

Cell death detection ELISA uncovered a remarkable apoptosis induction in MCF-7 and WEHI-164 cancer cells following exposure with DCM and MeOH extracts of *S. amplexicaulis*. Notably, the rate of apoptosis induced by DCM extract was much stronger than that caused by MeOH extract. Apoptotic DNA fragmentation detected by TUNEL assay also confirmed the results obtained from the ELSA. On the other hand, Western blot analysis exhibited that there was PARP cleavage in the cells treated with DCM and MeOH extracts. The nuclear enzyme PARP appears to be implicated in a variety of biological processes such as DNA repair, maintenance of genomic integrity and apoptosis. It is a 113 kDa protein that is cleaved by activated caspase-3 into two fragments of 89 and 24 kDa. This cleavage is considered as an important hallmark of cells undergoing apoptosis (Agarwal et al., 2009[1]; Pham et al., 2016[31]). These results are in line with the cytotoxicity data indicating *S. amplexicaulis* extracts as reliable apoptotic triggers.

Accumulated evidence demonstrated that p53 is a vital tumor suppressor gene that promotes cell cycle arrest and apoptosis. p53 can specifically activate expression of pro-apoptotic gene Bax and repress anti-apoptotic gene Bcl-2 (Al-Fatlawi et al., 2015[4]; Mitupatum et al., 2016[22]; Messeha et al., 2019[21]). The Bax gene product localizes to the mitochondria, mediates the permeabilization of the outer membrane, and enhances the release of cytochrome c into the cytosol, which leads to activation of caspase cascade gene and subsequent apoptosis (Peña-Blanco and García-Sáez, 2018[29]; Xiong et al., 2014[40]; Gheda et al., 2018[12]). In contrast, Bcl-2 gene encodes a protein that prevents apoptosis by binding and antagonizing with Bax (Al-Fatlawi et al., 2015[4]). Caspases belong to a large family of cysteine proteases that play crucial roles during the apoptotic process. Caspase 9 as an initiator caspase is involved in the activation of the executioner caspase-3 and the intrinsic (mitochondrial) pathway of apoptosis (Palai and Mishra, 2015[25]; Kalkhoran et al., 2017[17]). In the current study, we observed that treatment of cancer cells with DCM and MeOH extracts of *S. amplexicaulis* led to a decrease in the mRNA expression levels of Bcl-2 and increases in p-53, caspase-3, caspase-9, and Bax. These results suggest that *S. amplexicaulis* extracts can induce apoptosis through mitochondria mediated signaling pathway. Nevertheless, further investigations are needed to elucidate the detailed molecular mechanisms underlying the apoptotic activity of S*. amplexicaulis* extracts.

A remarkable number of anticancer compounds that have been commercially available in the market are derived from medicinal plants. Some cytotoxic compounds have been derived from the *Scrophularia* genus such as iridoid diglycoside, flavonoids, alkaloids, sugar esters and phenylpropanoid glycosides. Iridoid glycosides and their hydrolyzed compounds have exhibited anticancer properties against myeloid leukemia K562, gastric carcinoma MNK-45, and cervical carcinoma Hela cell lines. According to the phytochemical investigation, iridoid and phenylalkanoid glycosides have been identified in *S. amplexicaulis* and we presume that the cytotoxic activity of *S. amplexicaulis* may be attributed to the presence of these compounds; however, they should be explored in further studies.

In conclusion, our preliminary findings demonstrate that DCM and MeOH extracts of *S. amplexicaulis* are able to selectively kill cancer cells through induction of apoptosis in a mitochondrial intrinsic pathway. As a result, *S. amplexicaulis *medicinal plant can be considered as a promising chemotherapeutic candidate for extraction of anticancer components.

## Acknowledgements

The authors would like to thank the financial support of Immunology Research Center of Tabriz University of Medical Sciences.

## Conflict of interest

We declare that there is no conflict of interest.

## Figures and Tables

**Table 1:**
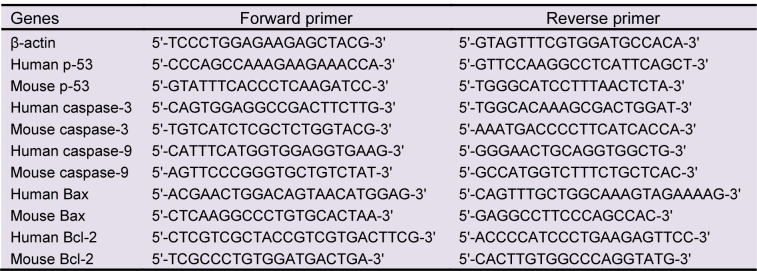
Primer squences used for Real-Time PCR

**Table 2:**
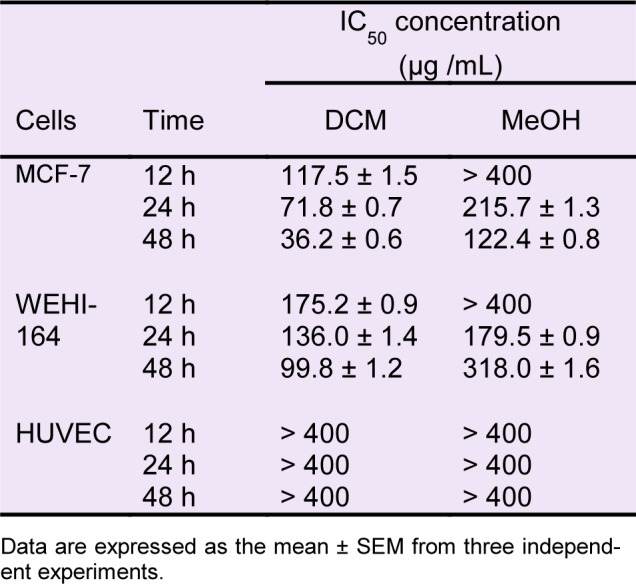
IC_50_ concentrations (µg/mL) of DCM and MeOH extracts of *S. amplexicaulis *in MCF-7, WEHI-164, and HUVEC cell lines
